# Effects of climate variability on the demography of wild geladas

**DOI:** 10.1002/ece3.8759

**Published:** 2022-03-26

**Authors:** Evan T. Sloan, Jacinta C. Beehner, Thore J. Bergman, Amy Lu, Noah Snyder‐Mackler, Hans Jacquemyn

**Affiliations:** ^1^ 26657 Plant Conservation and Population Biology Group Department of Biology KU Leuven Leuven Belgium; ^2^ 1259 Department of Psychology University of Michigan Ann Arbor Michigan USA; ^3^ 1259 Department of Anthropology University of Michigan Ann Arbor Michigan USA; ^4^ Department of Ecology and Evolutionary Biology University of Michigan Ann Arbor Michigan USA; ^5^ 12301 Department of Anthropology Stony Brook University Stony Brook New York USA; ^6^ 12301 Interdepartmental Program in Anthropological Sciences Stony Brook University Stony Brook New York USA; ^7^ 7864 School of Life Sciences Arizona State University Tempe Arizona USA; ^8^ 7864 Center for Evolution and Medicine Arizona State University Tempe Arizona USA

**Keywords:** climate change, demographic buffering, environmental stochasticity, primates, vital rates

## Abstract

Nonhuman primates are an essential part of tropical biodiversity and play key roles in many ecosystem functions, processes, and services. However, the impact of climate variability on nonhuman primates, whether anthropogenic or otherwise, remains poorly understood. In this study, we utilized age‐structured matrix population models to assess the population viability and demographic variability of a population of geladas (*Theropithecus gelada*) in the Simien Mountains, Ethiopia with the aim of revealing any underlying climatic influences. Using data from 2008 to 2019 we calculated annual, time‐averaged, and stochastic population growth rates (*λ*) and investigated relationships between vital rate variability and monthly cumulative rainfall and mean temperature. Our results showed that under the prevailing environmental conditions, the population will increase (*λ*_s_ = 1.021). Significant effects from rainfall and/or temperature variability were widely detected across vital rates; only the first year of infant survival and the individual years of juvenile survival were definitively unaffected. Generally, the higher temperature in the hot‐dry season led to lower survival and higher fecundity, while higher rainfall in the hot‐dry season led to increased survival and fecundity. Overall, these results provide evidence of greater effects of climate variability across a wider range of vital rates than those found in previous primate demography studies. This highlights that although primates have often shown substantial resilience to the direct effects of climate change, their vulnerability may vary with habitat type and across populations.

## Graphical abstract

This study used matrix population models and moving window climate analysis to explore the effects of rainfall and temperature variability on the vital rates of a population of geladas (*Theropithecus gelada*) in the Simien Mountains of Ethiopia. The higher temperature in the hot‐dry season generally led to decreased survival and increased fecundity, while higher rainfall in the hot‐dry season generally led to increased survival and fecundity. These widespread effects were contrary to prior similar primate research, highlighting that the vulnerability of primates to local climate change may vary with habitat type and population.

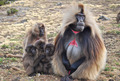

## 1 INTRODUCTION

Atmospheric concentrations of greenhouse gases are approaching levels unseen in the past 20 million years, critically altering the atmosphere, oceans, and freshwaters (Beerling & Royer, 2011). Concomitantly, temperature and precipitation regimes have become more variable and extreme and will likely continue to intensify globally (IPCC, 2021). Although the body of the literature regarding the effects of climate variability on animal demography and population dynamics continues to grow, the complexity of the underlying mechanisms demands further advances in research and methodology (Blois et al., 2013; Boyce et al., 2006). Climate changes will not, however, exert equivalent effects on all taxa, requiring detailed study to ensure that conservation challenges are met with a tailored approach (Moritz & Agudo, 2013). Nonhuman primates are among the most highly studied and most vulnerable mammal clades, with ~66.5% of species at risk of extinction (IUCN, 2021), but relatively few studies have investigated the effects of climate variability on their demography (Estrada et al., 2017). Although their frequent occurrence in forested habitats and small group sizes often present logistical and statistical obstacles to robust data collection and analysis, even scant demographic data have been shown to provide useful insight into the conservation needs of primate populations (Blomquist et al., 2009). Particularly in the current era of climate change, detailed demographic research can help disentangle the complex interactions between environmental factors and population shifts.

Previous studies have already indicated that climate can have pronounced effects on the demography of primates. For example, severe climatic events such as El Niño have been shown to greatly increase extinction risk in Milne Edward's sifaka (*Propithecus edwarsi*) populations already under pressure from hunting and deforestation (Dunham et al., 2008). Clear local climate effects on demography have also been found in Verreaux's sifakas (*Propithecus verreauxi*) (Lawler et al., 2009). Although studies such as these raise serious concerns given predicted increases in local climate extremes (Orlowsky & Seneviratne, 2012) and higher temperature and precipitation variance (Watterson, 2005) due to climate change, others have suggested substantial resilience of vital rates (which measure demographic rates such as survival, reproduction, and death) across a variety of species (Campos et al., 2017; Morris et al., 2011).

A study of seven primate species showed that populations tend to exhibit low temporal demographic variability compared to other vertebrate taxa and that this variability had little effect on long‐term fitness, suggesting that traits such as dietary flexibility, sociality, and highly developed cognition may buffer against the detrimental effects of environmental stochasticity (Morris et al., 2011). More comprehensive analyses of the same primate populations found relationships between local and/or larger‐scale climate variability and fecundity in three species, but little evidence for climate influences on survival (Campos et al., 2017). Notably, none of the vital rates for which climate variation was a strong predictor had large impacts on the population growth rate as a whole.

In this study, we used demographic and life history data to assess the viability and demographic variability of a gelada (*Theropithecus gelada*) study population with respect to underlying climate influences. Geladas aggregate into the largest groups of any nonhuman primate and are easily observed upon the Afro‐alpine grasslands they inhabit, which facilitates extensive data collection. Yet, their high altitude habitat and narrow, graminivorous diet may also make them particularly sensitive to climatic change and synergistic anthropogenic factors, though seasonal reliance on belowground plant organs during the dry season may buffer some of these effects (Fashing et al., 2014). Preliminary climate models have suggested that for every 2°C increase in mean global temperature, the lower altitudinal limit of gelada habitat will rise by 500 m and approximately halve the suitable habitat area (Dunbar, 1998). This pressure is likely to be exacerbated by the concurrent expansion of arable land. Sustained agricultural encroachment in protected areas has already led to severe erosion and significant loss of the aboveground biomass upon which geladas rely (Hunter, 2001). These pressures are corroborated by worsening trends of crop‐raiding and intensifying human–gelada conflict (Yihune et al., 2009). Furthermore, mounting research comparing study sites with different levels of anthropogenic disturbance supports the notion that these activities can significantly alter gelada feeding ecology and behavior (Fashing et al., 2014; Woldegeorgis & Bekele, 2015). These studies suggest that geladas may be under increasing threats, but detailed demographic analyses supporting these findings are currently lacking. In this study, we focused on identifying any significant effects of climate variability on vital rates by modeling vital rate responses to precipitation and temperature as well as assessing the viability of the study population, thus granting more detailed insight into how climate influences demographic change.

## 2 MATERIALS AND METHODS

### 2.1 Study species

Geladas are the lone extant species of the formerly widespread genus *Theropithecus* (Delson, 1993) and are endemic to the highlands of Ethiopia (Dunbar, 1993). Their range is often discontinuous as they only inhabit elevations from 1500 to 4500 m in which the rugged landscape is dominated by largely treeless plateaus of Afroalpine grassland fragmented by sheer escarpments (Iwamoto, 1993; Iwamoto & Dunbar, 1983). As the only graminivorous primate species, the gelada diet consists primarily of graminoid (grass‐like) leaves (Fashing et al., 2014; Hunter, 2001). Yet unlike other grazers, geladas organize into a complex, multi‐level social system of basic reproductive units of typically one male with 1–12 adult females and their offspring, bands of frequently associating units, and herds of converging bands reaching up to 1200 individuals (Kawai et al., 1983; Snyder‐Mackler et al., 2012). Geladas have been classified as moderately seasonal breeders (van Schaik et al., 1999) with an ecological birth peak occurring during maximum green grass availability in the late cold‐wet season; however, a larger social birth peak driven by seasonal male unit takeovers occurs in the cold‐dry season (Tinsley Johnson et al., 2018). Their uniquely compartmentalized social structure and terrestrial lifestyle facilitate demographic data collection despite very large group sizes. In addition, geladas have a dispersal pattern in which females remain with their natal unit through life while males emigrate to all‐male bachelor groups as subadults or even juveniles (Le Roux et al., 2011; Snyder‐Mackler et al., 2014). This dispersal system ensures a negligible amount of immigration and emigration by females which form the foundations of many demographic analyses.

At present, geladas are designated as Least Concern by the IUCN Red List as they are abundant within their range and no evidence suggests dramatic range‐wide decline (Gippoliti et al., 2019). Nonetheless, the species is protected under the Endangered Species Act and, like most primates, listed in Appendix II under the CITES treaty, which prohibits its trade for commercial purposes. Extrapolations from surveys in 1973 yielded a maximum range‐wide population estimate of 250,000 individuals (Dunbar, 1998), whereas the most recent surveys estimate a number closer to 50,000–60,000 individuals (J. C. Beehner & T. J. Bergman, unpublished data). With the global population in decline (Gippoliti et al., 2019) and all wild populations restricted to the Ethiopian plateau (Mori & Belay, 1990; Oates, 1996), these specialized primates are in a precarious position despite their relative stability.

### 2.2 Study site

**FIGURE 1:**
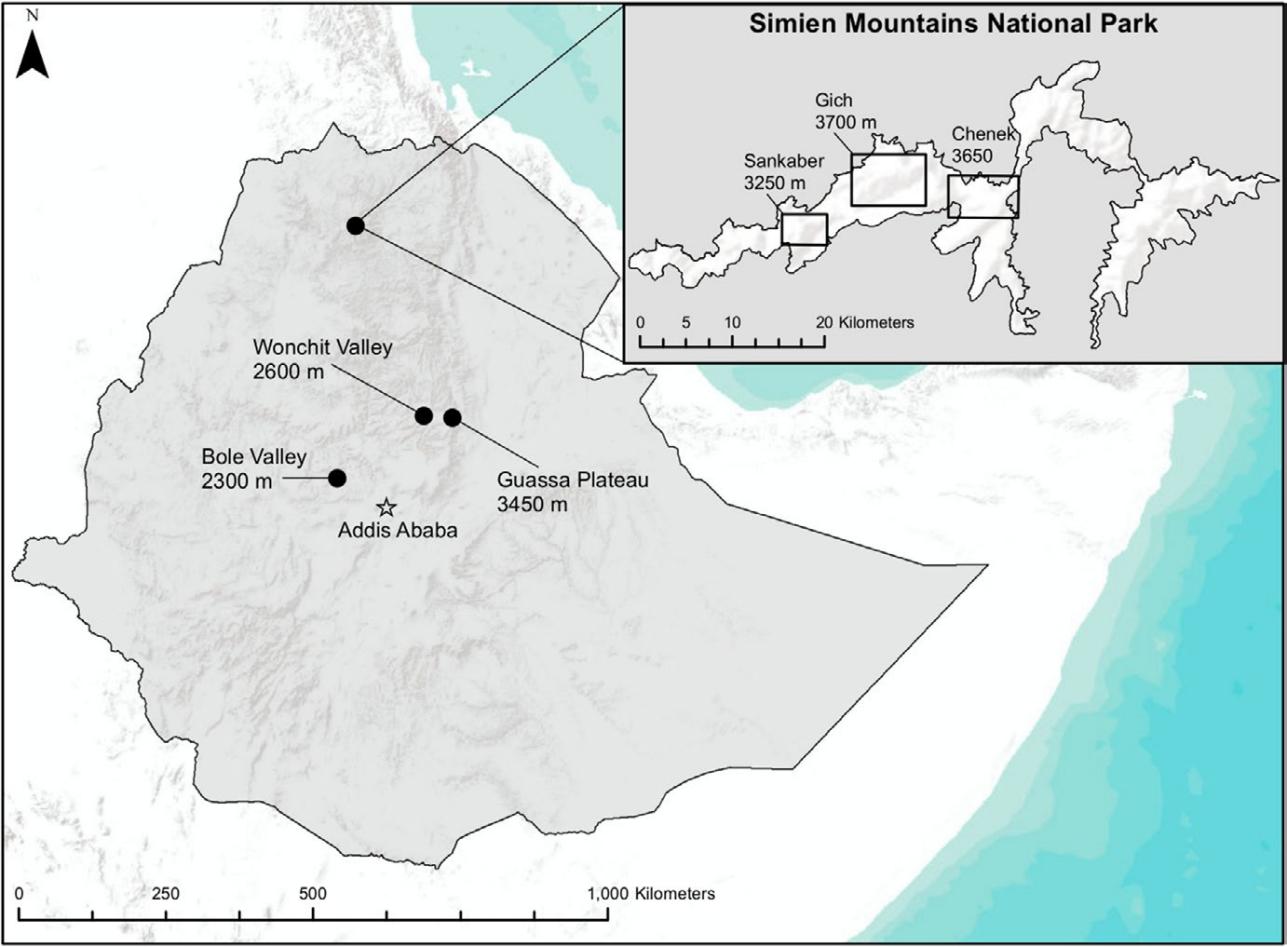
Location of Simien Mountains National Park and gelada populations throughout Ethiopia. Map sources: ESRI, Inc. 2016, Redlands, CA; Ethiopian Wildlife Conservation Authority

This study was conducted as part of the Simien Mountains Gelada Research Project in the Simien Mountains National Park in North Gondar, Ethiopia (Figure 1), which contains one of the largest populations of geladas (Beehner et al., 2007) and is the only area in which they are explicitly protected (Dunbar, 1993). In 2007, the Simien Mountains area was estimated to contain 4260–4560 geladas (Beehner et al., 2007); however, a more recent and comprehensive survey estimated this number closer to 7500 individuals (J. C. Beehner & T. J. Bergman, unpublished data). The study population inhabits the Sankaber region, which ranges from 13°12′40″ to 13°14′10″N and 38°00′47″ to 38°02′00″E with elevation from 3000 to 3300 m.

Sankaber and the Simien Mountains are identified as an Afroalpine grassland ecosystem (Puff & Nemomissa, 2005) characterized by dramatic escarpments topped with grassland and scrubland with sparse trees. Seasons are delineated by temperature and precipitation with the hot‐dry season running approximately from February to May, the cold‐wet season from June to September, and the cold‐dry season from October to January (Tinsley Johnson et al., 2018). Cold nights (<8.5°C) occur throughout the year, but daytime temperatures vary from average highs of ~16°C in the cold‐wet and cold‐dry seasons, to ~20.5°C in the hot‐dry season. Rainfall varies more dramatically throughout the year with a sharp peak during the cold‐wet season.

Despite its designation as a National Park in 1969, the Simien Mountains have historically suffered from a high amount of disturbance from agriculture (Dunbar, 1977; Iwamoto, 1979), and encroachment on protected land remains a prominent issue (Ejigu & Bekele, 2014; Woldegeorgis & Bekele, 2015). Levels of disturbance vary across Sankaber with human activity nominally prohibited on the plateau itself and increasing with proximity to the village of Michiby (also located within the park). Livestock encroachment was widespread prior to 2015 when new grazing restrictions went into place. In addition, a dirt and gravel road cuts through the study area and is often used to transport goods and people.

### 2.3 Data collection

Data were collected over 11 years between 31 December 2008 and 31 December 2019 from the Sankaber gelada population, using all female individuals from the units observed for the entire study duration. This included 281 total females and 128 females of reproductive age for estimating survival and fecundity rates, respectively. Behavioral, demographic, genetic, and hormonal data have been systematically collected by the project since 2006, but the routine recording of all demographic events for a core set of gelada reproductive units was not fully established until 2008. The dates for births, deaths, immigrations, emigrations, and developmental transitions were recorded as the first day the event was observed. If more than 2 days passed between observations of the corresponding group (and in the absence of other information such as a wet infant or an umbilical cord for births), we assigned the midpoint of the missing period as the event date. Deaths were assigned to females on the first day they were no longer observed with their reproductive unit. For females, this is appropriate because female geladas generally remain within their natal units throughout their lives. Similarly, we assumed that the disappearance of any infant prior to weaning was a case of mortality.

Daily weather data including cumulative rainfall (mm), minimum and maximum temperature (°C), and humidity were collected across the duration of the study period. This was respectively done using a rain gauge and a shaded La Crosse WS‐2315 U weather station (La Crosse Technology; La Crosse, WA, USA). Nearly 4 months of data were not collected in 2016 due to field site evacuation. While we could not interpolate these data, all other sporadic missing data over the study duration (due to occasional equipment failure or human error) were interpolated with a classification and regression tree (CART) machine learning algorithm using the *simputation* R package (van der Loo, 2021). CART algorithms use predictor values and cut points to split the sample into homogeneous subsamples, and form binary decision trees through this repeated process to impute the best values (Breiman et al., 1984).

### 2.4 Data analysis

#### 2.4.1 Population growth rates

The raw dataset contained comprehensive demographic and life history data from the study population, yielding 11 years of data to be split into separate 1‐year censuses taken on December 31 of each year. These were then transformed into corresponding projection matrices (**A**) populated with vital rates. Based on the minimum age at first reproduction of 4.9 years from a range of 4.9–7.6 years with a median of 6.1 (Roberts et al., 2017), there were five age classes before reaching adulthood (see Figure S1). We chose to use minimum age at first reproduction rather than alternatives such as median age as this more accurately reflected the gelada life cycle as well as numbers of adults and juveniles in each census.

As shown in the matrix below, *S*_n_, *S*_j_, and *S*_a_ represented the probability of survival from one census to the next for infants, juveniles, and adult females, respectively (i.e., the number of individuals in the age class at *t* + 1 divided by the number of individuals in the preceding age class at *t*). Fecundity (*F*_a_) was defined as the mean number of female offspring produced by all adult females across each intercensus interval with each adult capable of producing either 0 or 1 offspring. To determine the number of individuals that survived to the first age class in each year, fecundity (*F*_a_) was multiplied by the first infant survival rate (*S*_n1_), defined as the proportion of infants born in an intercensus interval that survived to the first census after their birth. Because these individuals were born at any time during the interval, they ranged from 1 day (if born on December 30th) to nearly 1 year (if born on January 1st) of age at the time of entering the first age class. The proportion of infants in the first age class that survived to become juveniles in the second age class was subsequently designated as the second infant survival rate (*S*_n2_). This transition (*S*_n2_) of infants to the juvenile stage emulated the median weaning age of 1.5 years with a range of approximately 1–2 years. Individuals then proceeded in an annual stepwise fashion through the four juvenile age classes before transitioning to the adult stage (*S*_j4_) at 5–6 years of age. The adult stage was not age‐stratified, so the matrix contained only one adult survival rate (*S*_a_) rather than proceeding through additional age classes. Please see the life cycle graph (Figure S1) in the Appendix S1 for full visualization and further explanation.

$$A\;=\;\left[\begin{array}{cccccc} 0 & 0 & 0 & 0 & 0 & S_{n1}\times F_{a}\\ S_{n2} & 0 & 0 & 0 & 0 & 0\\ 0 & S_{j1} & 0 & 0 & 0 & 0\\ 0 & 0 & S_{j2} & 0 & 0 & 0\\ 0 & 0 & 0 & S_{j3} & 0 & 0\\ 0 & 0 & 0 & 0 & S_{j4} & S_{a} \end{array}\right]$$

For each annual matrix, we calculated the dominant eigenvalue (*λ*) to assess the population growth rate as the population was close to a stable stage structure in each year. This was verified using Keyfitz’ Δ to compare the observed stage distribution of each annual matrix with the corresponding stable stage distribution (Keyfitz, 1968). These values ranged from 0.098 to 0.175, indicating that the study population remained close to a stable stage structure throughout the sampling period. In addition, we calculated the average deterministic growth rate and used numerical simulations to calculate the stochastic population growth rate (*λ*_s_) with 95% confidence intervals using the *stoch.growth.rate()* function with 50,000 iterations from the *popbio* package (Stubben & Milligan, 2007) in R (R Core Team, 2017).

In addition, sensitivities were calculated to determine the extent to which an absolute change in each vital rate leads to a change in *λ*. The relevant matrix elements were summed to acquire the cumulative sensitivities of infant and juvenile survival, while the chain rule was used to separate the sensitivity of the fecundity term from that of the first infant age class survival rate (Caswell, 2001).

#### 2.4.2 Climate analysis

Rainfall and temperature have long been established as important predictors of net primary productivity (NPP), which further correlates to plant biomass (Chu et al., 2016). By extension, they are likely a reasonable metric for food availability, which inevitably affects vital rates. Prior research on gelada feeding ecology in the Simien Mountains has also found that aboveground food availability strongly positively correlated with rainfall across the previous 30, 60, and 90 days (Jarvey et al., 2018). To be conservative, we tested both cumulative monthly rainfall and mean monthly temperature for their relationships to vital rates.

This was accomplished with a moving window approach using the *climwin* package (Bailey & van de Pol, 2016) to assess the effects of climate on gelada vital rates across every possible combination of consecutive time windows within the 24 months preceding each census on December 31. In keeping with Campos et al. (2017), a 24‐month timeframe was chosen to accommodate the lagged environmental effects on demography that have been shown to occur in primates (Wiederholt & Post, 2011) and other animals (Hansen et al., 2013). This method did, however, exclude the 2016 and 2017 censuses due to missing data. We then converted each vital rate into a binary list in which each relevant individual was assigned either a 1 if they survived to the next census or gave birth in the intercensus interval or a 0 otherwise. These were then modeled in generalized linear mixed models (GLMMs) with binomial error distributions and a yearly random effect on the mean vital rate to investigate potential relationships between vital rates and variability in either cumulative monthly rainfall or average monthly temperature. Collinearity was avoided by the inclusion of only one climate variable per model.

For each combination of vital rates and climate variables, models of each time window were ranked by sample size‐corrected Akaike's information criterion (AIC_C_; Burnham & Anderson, 2002) against a null model with no climate variable. The moving window approach suffers from a relatively high risk of false positives due to the sheer number of models involved. Subsequently, *P*_C_ statistics have been shown to effectively discriminate Type I and Type II errors from true climate signals in as few as five iterations (van de Pol et al., 2016). These values were determined by comparing the number of models from observed versus randomized data falling into the 95% confidence set over 100 iterations (van de Pol et al., 2016). Each *P*_C_ < 0.5 was then subjected to *k*‐fold cross‐validation to further evaluate model validity. *k* should ideally be a whole number divisor of the number of trials, so with 9 years of data, *k* = 3 was chosen. Model reliabilities were further tested by evaluating the quantity of model weights falling within the 95% confidence set where a lower percentage indicates higher confidence in a true signal (Bailey & van de Pol, 2016). The strength of the relationship between a given climate variable and the vital rate was represented by *β*, which signifies the degree to which change in the climate variable corresponds to change in the vital rate.

Because our dataset does not have the necessary level of sampling variability, we did not estimate annual vital rates based on GLMMs with the random effect of year to reduce sampling error. Other studies that have done so had some level of sampling variability inherent to their methodology. For example, (Altwegg et al., 2007) acquired survival data on barn owls (*Tyto alba*) through a mark‐recapture method, and Morris et al. (2011) used both males and females to calculate survival rates, thus introducing sampling variability due to the conflation of dispersal and mortality for either males or females. On the contrary, geladas have a matrilineal social system (i.e., females do not disperse) and we exclusively used data collected through direct observation of female individuals. Disappearances could therefore always be considered mortality, particularly as they were almost always preceded by injury or disease.

## 3 RESULTS

### 3.1 Descriptive statistics, population growth rates, and other life history statistics

The sample population of females consisted of 281 unique individuals. Of 331 births, 157 were females, 174 were males, and 3 were unsexed, which yields an even sex ratio of 47% females to 53% males. Deaths included three unsexed infants, 10 female infants, 53 juvenile females, and 56 adult females.

**FIGURE 2:**
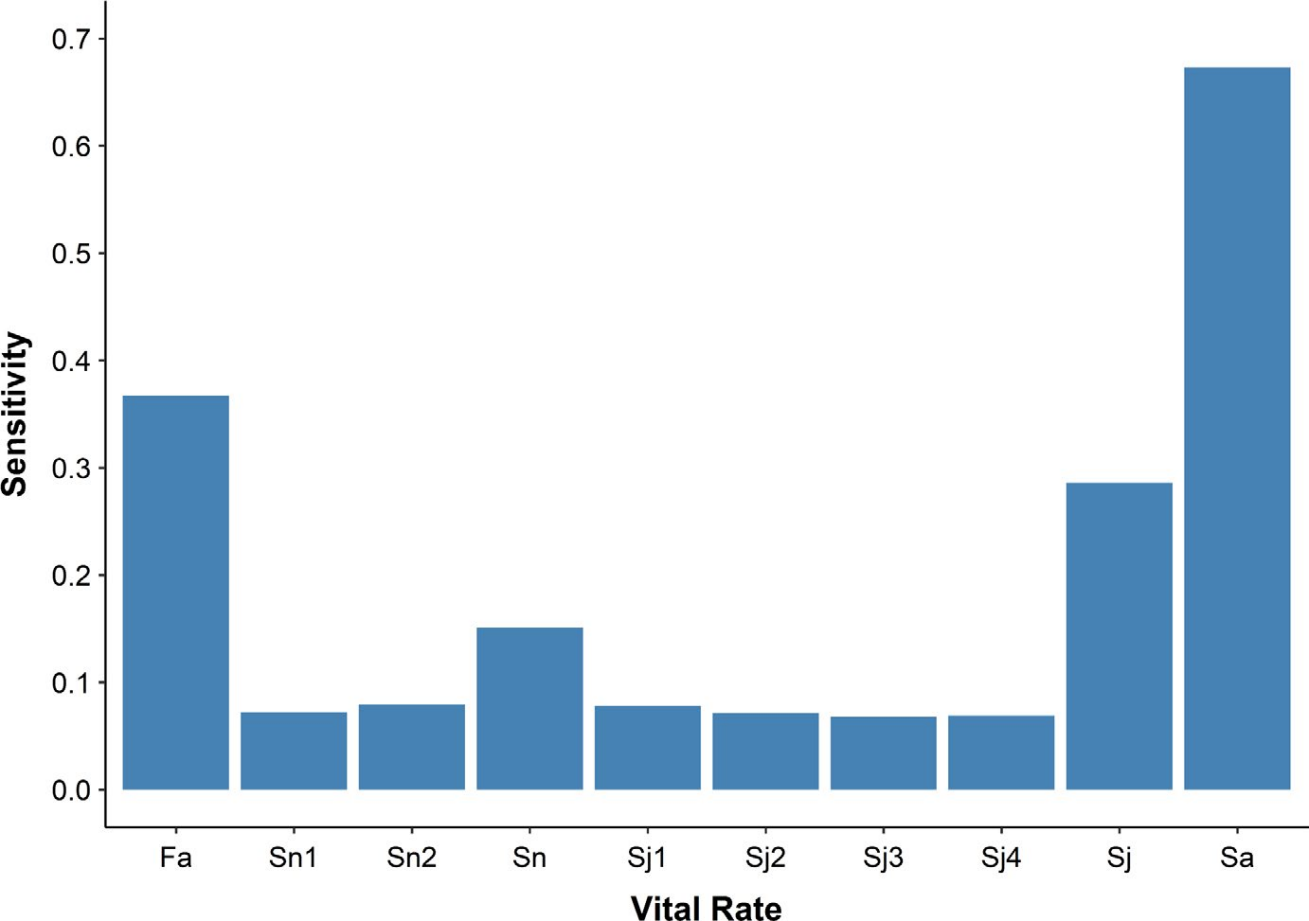
Time‐averaged sensitivities of population growth rate (*λ*) to each vital rate wherein higher values represent greater influence upon *λ*. *S*_n_, *S*_j_, and *S*_a_ are respectively infant, juvenile, and adult survival. *S*_n_ and *S*_j_ are further split into their respective age classes. *F*_a_ is adult fecundity

Deterministic population growth rates varied between 0.9427 and 1.0607 (Table 1) with an average of 1.0218. The corresponding stochastic growth rate (*λ*_s_) was 1.0208. A time‐averaged matrix was also used to compute net reproductive rate (1.43 individuals) and female lifespan conditional upon survival to adulthood (17.8 years). Net reproductive rate is defined as the average number of female offspring born to each female during her lifespan. The population growth rate was consistently most sensitive to adult survival, moderately sensitive to fecundity and juvenile survival, and least sensitive to infant survival (Figure 2). In addition, it was comparably sensitive to each individual age class of both infant and juvenile survival.

**TABLE 1:** Population growth rate (*λ*) and sample sizes in each annual census period and the aggregated stochastic growth rate (*λ*_S_)

Population growth rates (*λ*)												
Year	08.09	09.10	10.11	11.12	12.13	13.14	14.15	15.16	16.17	17.18	18.19	Stoch
*λ*	0.9787	0.9902	1.0391	1.0132	1.0091	1.0607	1.0584	1.0043	1.0389	0.9427	1.0540	1.0208
*N*	125	127	124	124	133	138	144	150	164	156	149	N/A

Note

*λ* ranged between 0.9427 in 2017 to 2018 and 1.0607 in 2013 to 2014.

### 3.2 Climate analysis

**FIGURE 3:**
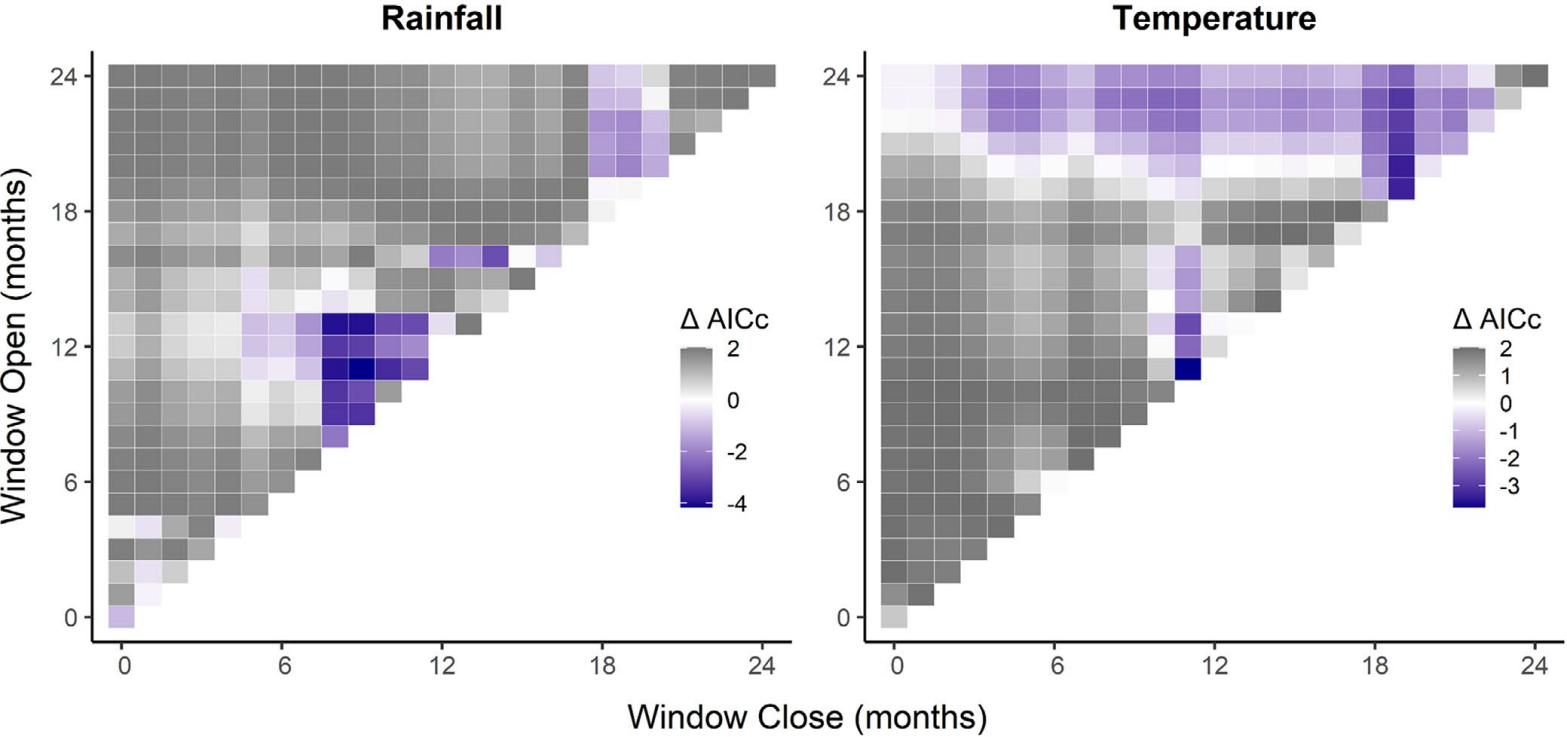
Moving window analysis of ΔAICc for the effect of cumulative monthly rainfall and monthly mean temperature on adult fecundity. Each grid cell corresponds to a time window that opens and closes between 0 and 24 months before the annual census as indicated on the axes. Deeper purple cells indicate more informative models relative to a null model without climate variables

Moving window climate analysis showed fecundity to have significant positive relationships with both rainfall (*P*_C_ = 0.460) and temperature (*P*_C_ = 0.445). In the best models, the rainfall from February to April (hot‐dry season) of the census year (ΔAIC_C_ = −4.181, Figure 3) and the temperature in February of the census year (ΔAIC_C_ = −3.804, Figure 3) had positive effects on fecundity (*β* = 0.346 and 0.439, respectively).

**FIGURE 4:**
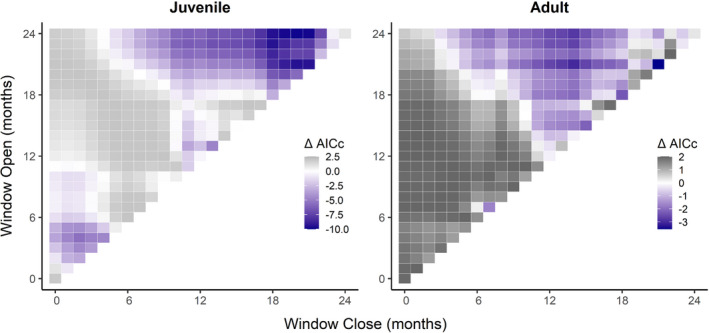
Moving window analysis of ΔAICc for the effect of mean monthly temperature on survival of juveniles and adults. Each grid cell corresponds to a time window that opens and closes between 0 and 24 months before the annual census as indicated on the axes. Deeper purple cells indicate more informative models relative to a null model without climate variables

Of the four juvenile survival rates, none were significantly affected by climate influences; however, when aggregated into a single vital rate to evaluate the unified juvenile life stage and account for low sample sizes, there was a significant relationship with temperature (*P*_C_ = 0.064). The best model included January to May of the year prior to the census year (ΔAIC_C_ = −9.847, Figure 4), indicating that higher temperature in the hot‐dry season of the previous year may decrease survival in the subsequent year (*β* = −3.230).

**FIGURE 5:**
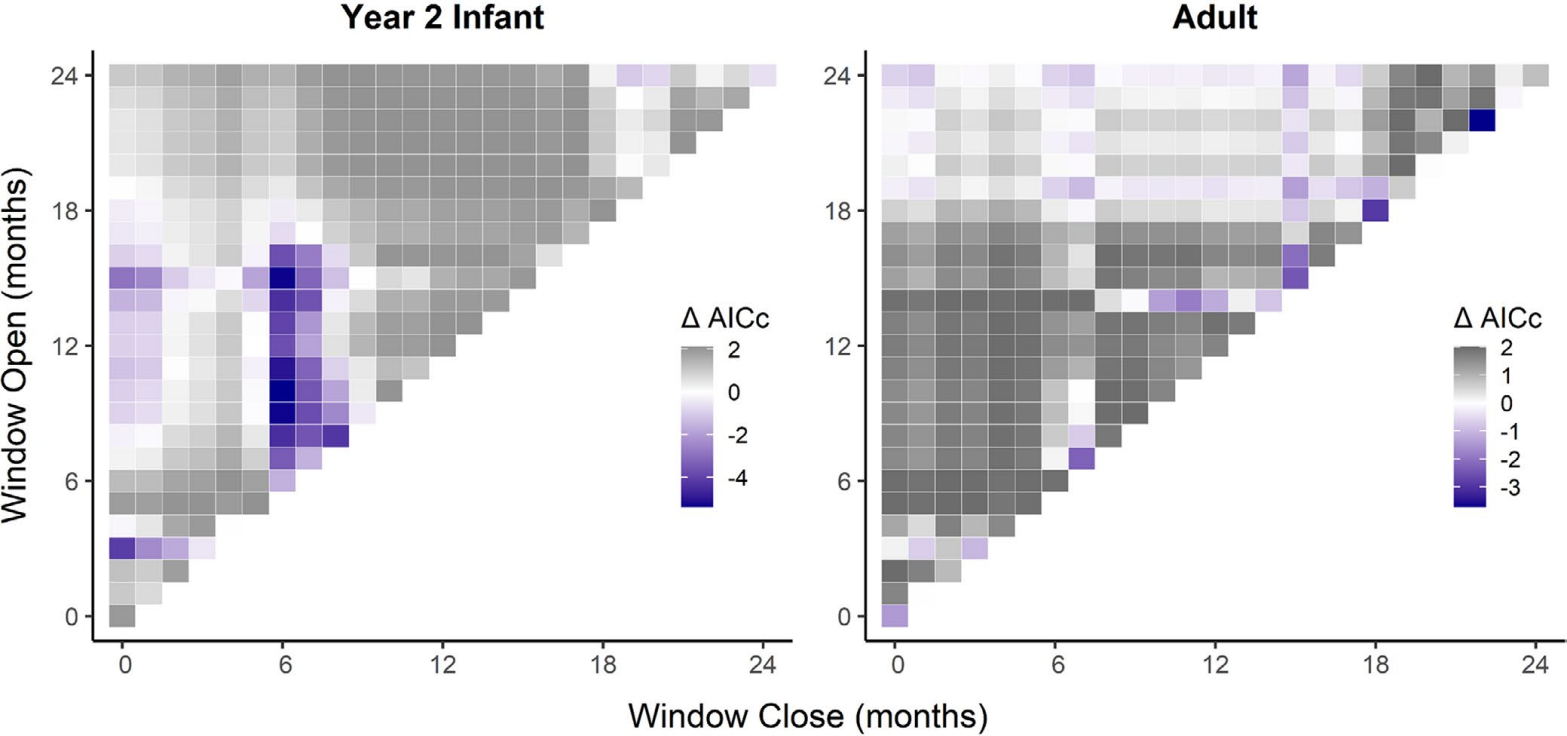
Moving window analysis of ΔAICc for the effect of cumulative monthly rainfall on survival of the second infant age class and adults. Each grid cell corresponds to a time window that opens and closes between 0 and 24 months before the annual census as indicated on the axes. Deeper purple cells indicate more informative models relative to a null model without climate variables

Adult survival had significant relationships with both temperature (*P*_C_ = 0.429) and rainfall (*P*_C_ = 0.495). In the best models, the temperature in April of the previous year (ΔAIC_C_ = −3.554, Figure 4) and the rainfall in March of the previous year (ΔAIC_C_ = −3.715, Figure 5) had negative relationships to adult survival (*β* = −0.523, *β* = −0.607). These results indicate that higher temperature and rainfall in the hot‐dry season may decrease survival in the following year. Conversely, the second infant survival rate (*S*_n2_) had a significant positive relationship with rainfall (*P*_C_ = 0.450) with the best model including March to July of the census year (ΔAIC_C_ = −5.378, Figure 5), which suggests that higher rainfall in the hot‐dry season and early cold‐wet may lead to higher infant survival (*β* = 0.896). For the top models of all combinations of vital rates and climate variables with corresponding statistics, see Table S1.

## 4 DISCUSSION

### 4.1 Population growth rates

Although the results indicate that the average population growth rate of the Sankaber gelada population was >1, the annual growth rates varied greatly over the study period from 0.9427 to 1.0607. The sensitivity analysis showed that adult survival had the greatest influence on the population growth rate, while infant survival had the least influence (Figure 2). The population growth rate was moderately sensitive to fecundity and, slightly less so, to juvenile survival. This largely corroborates the generally held expectations for long‐lived vertebrates (Eberhardt, 2002) including primates (Lawler, 2011; Morris et al., 2011). Furthermore, Pfister’s (1998) hypothesis proposes that vital rates which strongly influence fitness should vary less over time. This is based on the prediction that increasing vital rate variation decreases long‐term fitness; therefore, natural selection should act to reduce variability in vital rates with the greatest influence on population growth rate (Hilde et al., 2020). Visual comparisons of sensitivities to variance largely aligned with this predicted pattern (see Figure S2).

With a demographic stochastic growth rate of 1.0208 ± 0.0003, one would expect increasing population size over time; however, considering this calculation within a broader spatiotemporal scope may grant some insight into the dynamics of the greater Simien Mountains metapopulation. Censuses over an approximately 30‐year period have shown that the population at Sankaber as well as those at two other sites (Michibi and Gich) have remained stable across this period (Beehner et al., 2007; Dunbar & Dunbar, 1975; Ohsawa, 1979). These areas are fully within the national park boundaries and are more heavily monitored than external areas. Though there has been little systematic research on the protective influence of active scientific field stations, research presence may provide some buffering against human encroachment (Laurance, 2013). These routinely monitored populations could therefore be more insulated against anthropogenic disturbance than other less protected areas within and outside the park boundaries. Especially given evidence for worsening anthropogenic threats such as erosion and loss of aboveground biomass due to agricultural activity (Hunter, 2001) and increased rates of crop‐raiding (Yihune et al., 2009), stable population size in certain insulated areas fits neatly into a framework of source‐sink dynamics (Dunning et al., 1992; Gundersen et al., 2001).

### 4.2 Demographic variability versus climate variability

The moving window analyses detected effects of rainfall and temperature in many vital rates in the Sankaber gelada population, including fecundity and adult, combined juvenile, and second infant survival rates. The latter had a positive relationship to rainfall, indicating that higher rainfall in the hot‐dry season and early cold‐wet season was linked to increased survival in the second infant age class (*β* = 0.896). Peak green grass availability typically ends in the cold‐dry season, with worsening food scarcity persisting through the hot‐dry season until rainfall begins to increase during the cold‐wet season of the following year. During this time, geladas increasingly rely on subterranean foods such as roots, corms, and tubers (Fashing et al., 2014; Iwamoto, 1979) whose energetic returns on investment may be limited by the time and effort of their harvest (Hunter, 2001). Perhaps if young geladas at the weaning transition do not access underground foods with the same efficiency as adults, the more rapid return to green grass availability due to dry‐season rains increases their ability to survive the dry season.

Juveniles across several primate taxa have been shown to exhibit lower foraging efficiency and, in some cases, higher mortality in times of food scarcity (Janson & van Schaik, 2002). Furthermore, these effects typically diminish rapidly with age (Janson & van Schaik, 2002), supporting the premise that such impacts would be particularly salient in young individuals experiencing their first trials of independently foraging subterranean foods. This prior research is supported by the disappearance of associated climate effects in the survival rates of all subsequent juvenile age classes. Similar patterns of vulnerability to rainfall‐linked environmental factors in recently independent individuals have also been substantiated by prior demographic studies on Verreaux's sifaka (Lawler et al., 2009; Richard et al., 2000) and rhesus macaques (*Macaca mulatta*) (Blomquist, 2013).

While effects of rainfall were detected in the second infant survival rate, the first infant survival rate corresponded to no detectable climate effects. In addition, first infant and adult survival rates had moderate covariance (0.473), whereas no such relationship occurred between second infant and adult survival rates. This aligns with infant development patterns in which older infants will have started to become increasingly independent from their mothers and more reliant on foraged foods due to the intensification and completion of the weaning process during this period. Conversely, the covariance between first infant and adult survival corroborates prior research that maternal disappearance or death comprised a major source of infant mortality in geladas (Beehner & Bergman, 2008). Furthermore, in the Sankaber population, infants and lactating mothers suffered significantly higher rates of injury during the three months following a takeover (Schneider‐Crease et al., 2020). Nearly 60% of all infant mortality was attributed to infanticide and the likelihood of death was 32 times higher in the four months following a unit takeover than in periods without takeovers (Beehner & Bergman, 2008). These data suggest that the demographic upheaval brought about by dramatic social events such as male takeovers can overshadow any effects of climate variability in certain vital rates.

With that said, the effects of climate variability during the dry season were prevalent. Fecundity showed a positive relationship to rainfall during the hot‐dry season (*β* = 0.346). Although prior research did not find any links between green grass availability and fecundity, an energetic benefit from earlier or greater green grass availability nonetheless seems intuitively probable (Tinsley Johnson et al., 2018). In addition, higher fecundity most strongly correlated with higher temperatures in the early dry‐season of the census year (*β* = 0.439). This aligns with known correlations linking higher temperatures to lower glucocorticoid levels and returns to reproductive cycling (Tinsley Johnson et al., 2018), which were particularly prevalent in the hot‐dry season. Furthermore, the strongest model window fell within the takeover season during which the majority of all such events occur (Pappano & Beehner, 2014) and returns to cycling would be most expected (Tinsley Johnson et al., 2018).

Combined juvenile survival had clear evidence of climate signals, indicating that higher hot‐dry season temperature in the preceding year was linked to lower survival (*β* = −3.230). No climate signals were detected in the survival rates of the individual juvenile age classes, but their much smaller sample sizes in each year may have contributed to this absence. A similar, albeit much weaker, the relationship was also found in adult survival (*β* = −0.523). These relationships could be related to heat stress, but prior research suggests that thermoregulatory stressors in geladas are typically associated with cold temperatures (Dunbar, 1980; Tinsley Johnson et al., 2018). However, there may also be more opaque, indirect explanations for these lagged effects. Although 1‐year lagged effects have only been shown in primates due to rainfall and the El Niño climate oscillation (*Brachyteles hypoxanthus* and *Lagothrix lagothricha*: Wiederholt & Post, 2011), lagged effects from temperature have been found in a variety of montane and grassland mammals including on population growth in American bison (*Bison bison*: Koons et al., 2015) and adult female survival and juvenile recruitment in woodland caribou (*Rangifer tarandus caribou*: DeMars et al., 2021). Willisch et al. (2013) found that higher winter air temperatures corresponded to decreased survival in adult males and yearlings in alpine chamois (*Rupicapra rupicapra*). Counterintuitive results such as these and the relationship between temperature and juvenile and adult survival in our study suggest that such effects likely arise from indirect and density‐dependent factors such as competition and community‐level interactions. For example, the demonstrated temperature‐induced increases in fecundity may increase competition for resources in the following year. Density‐dependent factors could also be of particular importance given that resource availability, and therefore competition undergoes dramatic seasonal variation compared to tropical environments. While we do not yet understand how long‐term climatic cycles affect the availability of grass and other resources in Afroalpine grasslands, warmer temperatures have been shown to exert negative effects on the phenology of numerous grassland plant species in the year after they occur in American tallgrass prairie (Sherry et al., 2011). Should such lagged phenological effects occur, research on female olive baboons (*Papio anubis*) has shown that the intensity of intragroup feeding competition increased during times of lower food availability and higher reliance on fallback foods (Barton & Whiten, 1993; Johnson, 1989), potentially influencing survival. Although this study cannot explain the complex underlying mechanisms by which climate variability influences demographic rates, it nonetheless highlights the risks geladas face as trends in climate change continue to worsen.

Lastly, higher rainfall in the hot‐dry season of the previous year correlated to lower adult survival (*β* = −0.196). No suitable explanation could be found for this relationship and given the strong links between rainfall and NPP, it seems unlikely that higher rainfall during a period of food scarcity would cause a decrease in survival rate. Furthermore, the very weak and inconsistent model weight distribution of this relationship gives reason for skepticism.

### 4.3 Comparison to other primates

Mean vital rates from the Sankaber gelada population largely corroborate the analyses of seven other primate species (Morris et al., 2011). Nearly all of the species, including geladas, were characterized by high survivorship throughout all life stages and relatively low fecundity as expected from the typical reproductive strategy of primates. However, widespread evidence for climate effects across vital rates distinguished geladas from other primates in which similar research has been done. Of seven other primate species previously analyzed using similar modeling techniques, four showed evidence of climate influences upon vital rates, but among these only Verreaux's sifaka did so for more than one vital rate (Campos et al., 2017). In addition, the strongest effects were seen in the three most highly seasonal breeders: Verreaux's sifaka, blue monkeys (*Cercopithecus mitis*), and northern muriquis (*Brachyteles hypoxanthus*). Geladas are moderately seasonal breeders (van Schaik et al., 1999); however, the larger of two distinct birth peaks occur in accordance with social rather than environmental drivers (Tinsley Johnson et al., 2018). The aforementioned study found no evidence of climate effects in the moderately seasonal white‐faced capuchins (*Cebus capucinus*) or yellow baboons (*Papio cynocephalus*) (Campos et al., 2017), indicating that this level of reproductive seasonality alone may be insufficient to cause salient relationships between climate and vital rates. Nonetheless, our study did provide evidence for influences of temperature variability on fecundity, suggesting that although reproductive seasonality is predominantly dictated by social factors such as unit takeovers, there may be environmental interactions.

Why then might geladas have greater evidence of climate influences across a wider range of vital rates compared to these previously studied primates? The vast majority of primates inhabit tropical ecosystems characterized by highly variable intra‐ and interannual rainfall patterns (Feng et al., 2013), whereas geladas and the small number of temperate species, including snub‐nosed monkeys (*Rhinopithecus* spp.) and Japanese macaques, further experience substantial daily and seasonal variation in temperature which impose additional thermoregulatory and energetic constraints. Cold temperatures have been shown to present obstacles to female reproduction in geladas (Tinsley Johnson et al., 2018) and other primates (*Aotus azarai*: Fernandez‐Duque et al., 2002; *Cercopithecus mitis*: Foerster et al., 2012; *Rhinopithecus bieti*: Xiang & Sayers, 2009), as well as increased mortality in both geladas (Dunbar, 1980) and Japanese macaques (Enari, 2014). Temperature extremes likely also increase the importance of accessibility and quality of fallback foods as previously shown in both Japanese macaques (Hanya et al., 2006) and black and white snub‐nosed monkeys (Grueter et al., 2009). Although we did not reveal these specific relationships, these examples illustrate that temperature extremes greatly influence the life history events of primates inhabiting such ecosystems and support the particular importance of temperature as shown by the salient effects on fecundity and adult and juvenile survival found in this study. Given the demonstrated responses of gelada vital rates and the general sensitivity of montane environments to climate change, elucidating the causal relationships between these unique environmental conditions and demography will only become increasingly pertinent. Nonetheless, a deeper understanding will require additional research on geladas and other cold‐weather primates.

## 5 CONCLUSIONS AND FUTURE DIRECTIONS

This study revealed more extensive climate influences across vital rates in geladas compared to prior primate studies. Although the results painted a tentatively positive picture of a healthy and stable study population, it also revealed the need for a more thorough assessment of the broader Simien Mountains metapopulation and geladas as a whole. This is particularly pertinent given evidence for the sensitivity of geladas to climate change due to the environmental extremes of their habitat and the apparent strong responses of their vital rates to changes in climatic conditions. The study also highlighted a continued mismatch between demographic studies suggesting resilience to climatic change (Campos et al., 2017) and the grimmer conclusions of studies investigating phenomena such as extreme climate events and broad‐scale climate change (Estrada et al., 2017; Korstjens & Hillyer, 2016; Zhang et al., 2019).

This study did not investigate social factors, but prior research has widely demonstrated their great importance. In particular, takeovers have been shown to significantly influence conceptions and births (Tinsley Johnson et al., 2018) and adult female and infant mortality (Schneider‐Crease et al., 2020). Future research should therefore explore the impact of such events to provide a more complete understanding of gelada demographic variation. An understanding of how environmental variability affects takeover frequency and timing would improve not only an understanding of the mechanistic drivers of these social shifts but also the ability to predict their frequency and the demographic changes that follow. These social dynamics may also be more broadly linked to density‐dependent factors such as resource competition that result from climate variability and other environmental factors.

In conclusion, further research should aim to disentangle the network of influences from direct climate variability, indirect climate change synergies resulting in range loss, stochastic severe weather events, and indirect climate effects acting through social dynamics. This information will benefit not only geladas but also the greater primate order and other organisms with similar life‐history strategies, particularly in the current era of accelerating climate change.

## CONFLICT OF INTERESTS

The authors declare no conflicts of interest.

## AUTHOR CONTRIBUTIONS

**Evan Taylor Sloan:** Conceptualization (lead); Data curation (equal); Formal analysis (lead); Investigation (lead); Methodology (equal); Writing – original draft (lead); Writing – review & editing (equal). **Jacinta Beehner:** Data curation (equal); Funding acquisition (equal); Project administration (equal); Resources (equal); Supervision (equal); Writing – review & editing (equal). **Thore Bergman:** Data curation (equal); Funding acquisition (equal); Project administration (equal); Resources (equal); Writing – review & editing (equal). **Amy Lu:** Data curation (equal); Funding acquisition (equal); Project administration (equal); Resources (equal); Writing – review & editing (equal). **Noah Snyder‐Mackler:** Data curation (equal); Funding acquisition (equal); Project administration (equal); Resources (equal); Writing – review & editing (equal). **Hans Jacquemyn:** Conceptualization (supporting); Formal analysis (supporting); Investigation (supporting); Methodology (equal); Supervision (equal); Writing – original draft (supporting); Writing – review & editing (equal).

## Supporting information

Supplementary Material

## Data Availability

Data and R scripts are available through the Simien Mountains Gelada Research Project GitHub repository: https://github.com/GeladaResearchProject/Sloan_et_al_2022‐climate‐demography.
