# Supplementary material for: Effects of climate variability on the demography of wild geladas
Source: Ecol Evol. 2022 Mar 26;12(3):e8759. doi: 10.1002/ece3.8759 (PMC8956858; doi:10.1002/ece3.8759)
Supplement: Supplementary file 1 — Supplementary Material [file ECE3-12-e8759-s001.docx]

**Appendix**


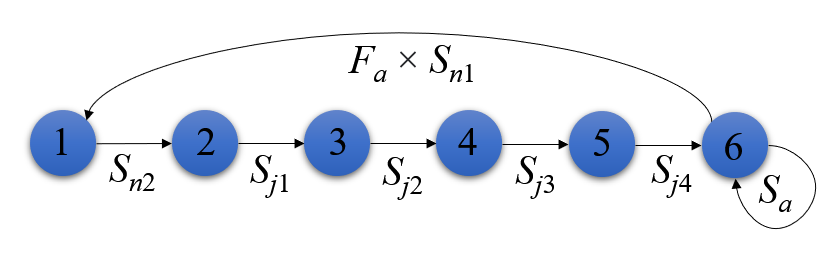


**Fig. S1** Life cycle graph illustrating life stages and vital rates. *S_n_, S*_j_, and *S*_a_ represent the probability of survival from one census to the next for infants, juveniles, and adult females, respectively (i.e., the number of individuals in the age class at *t* + 1 divided by the number of individuals in the preceding age class at *t*). To determine the number of individuals that survived the first age class in each year, fecundity (*F*_a_) was multiplied by the first infant survival rate (*S_n1_*), defined as the proportion of infants born in an intercensus interval that survived to the first census after their birth. Because these individuals were born at any time during the interval, they ranged from 1 day to nearly 1 year of age at the time of entering age class 1. For example, if an individual is born in January and survives to their first census (*S_n1_*), thus entering age class 1, they will already be nearly 1 year old, so if they survive to age class 2, they will be nearly 2 years old. Conversely, if an individual is born in December and survives to their first census, they will be less than 1 month old, so by the time they reach age class 2, they will be slightly over 1 year old. The proportion of infants in age class 1 that survived to become juveniles in age class 2 was subsequently designated as the second infant survival rate (*S_n2_*). This transition (*S_n2_*) of infants to the juvenile stage emulated the geladas median weaning age of 1.5 years with a range of approximately 1 to 2 years. Individuals then proceeded in an annual stepwise fashion through the four juvenile age classes (2 to 5) before transitioning to the adult stage (*S_j4_*) at 5 to 6 years of age. The adult stage (age class 6) was not age-stratified, so the matrix contained only one adult survival rate (*S_a_*) rather than proceeding through additional age classes. The 1 year age range within each cohort is an inevitable byproduct of matrix construction based on fixed 1 year censuses, but in our case, it adequately approximates the biological reality of this species in both weaning and maturation age.


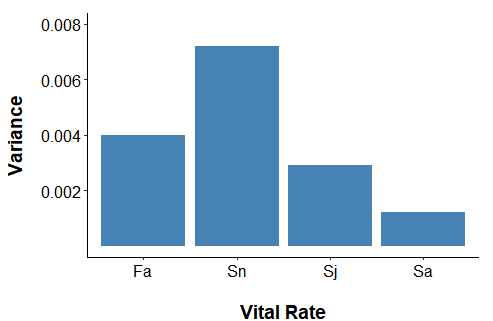


**Fig. S2** Variance of vital rates to illustrate Pfister’s hypothesis that vital rates with higher sensitivities have lower variance. S_n_, S_j_, and S_a_ are respectively infant, juvenile, and adult survival. S_n_ and S_j_, while F_a_ is adult fecundity. As discussed in the main text, population growth rate was most sensitive to adult survival, moderately sensitive to fecundity and juvenile survival, and least sensitive to infant survival (Fig. 2). According to these sensitivities the variances shown above support Pfister’s hypothesis.

**Table S1.** Top models for all combinations of vital rates and climate variables. Note that “Month Open” and “Month Closed” signify the number of the months before the annual census on December 31 to a maximum of 24 months. P_c_ < 0.5 (marked ‘*’) was significant. S_n_ and S_j_ represent the aggregated infant and juvenile vital rates, respectively. S_j3_ could not produce valid results as no deaths occurred in this vital rate during any of the analyzed years.

| **Vital Rate** | **Variable** | **Month Open** | **Month Closed** | **ΔAICc** | **β** | **Std. Error** | **P_c_ Value** |
| --- | --- | --- | --- | --- | --- | --- | --- |
| S_n1_ | Rainfall | 15 | 6 | 1.899 | 0.056 | 0.112 | 0.561 |
|  | Temperature | 7 | 7 | 1.883 | -0.068 | 0.131 | 0.561 |
| S_n2_ | Rainfall | 10 | 6 | -5.378 | 0.896 | 0.352 | 0.450* |
|  | Temperature | 7 | 7 | -3.634 | -1.324 | 0.565 | 0.586 |
| S_n_ | Rainfall | 10 | 6 | 1.143 | 0.055 | 0.057 | 0.554 |
|  | Temperature | 7 | 7 | 1.214 | -0.095 | 0.102 | 0.561 |
| S_j1_ | Rainfall | 14 | 5 | 1.004 | -0.133 | 0.125 | 0.553 |
|  | Temperature | 22 | 17 | 0.665 | -0.406 | 0.340 | 0.549 |
| S_j2_ | Rainfall | 18 | 18 | 1.910 | 0.028 | 0.047 | 0.561 |
|  | Temperature | 22 | 9 | 1.887 | -0.232 | 0.380 | 0.557 |
| S_j3_ | Rainfall | - | - | - | - | - | NA |
|  | Temperature | - | - | - | - | - | NA |
| S_j4_ | Rainfall | 7 | 6 | 2.160 | 0.010 | 0.046 | 0.561 |
|  | Temperature | 14 | 14 | 2.156 | -0.068 | 0.301 | 0.561 |
| S_j_ | Rainfall | 14 | 4 | -6.064 | -1.099 | 0.411 | 0.558 |
|  | Temperature | 24 | 20 | -9.847 | -3.230 | 1.157 | 0.064* |
| S_a_ | Rainfall | 22 | 22 | -3.715 | -0.607 | 0.241 | 0.495* |
|  | Temperature | 21 | 21 | -3.554 | -0.523 | 0.228 | 0.429* |
| F_a_ | Rainfall | 11 | 9 | -4.181 | 0.346 | 0.136 | 0.473* |
|  | Temperature | 11 | 11 | -3.804 | 0.439 | 0.182 | 0.445* |
